# Plastic and Reconstructive Surgery in the Era of Artificial Intelligence

**DOI:** 10.1055/s-0046-1824538

**Published:** 2026-06-23

**Authors:** Samreen Jaffar, Maneesh Singhal

**Affiliations:** 1 Department of Plastic and Reconstructive Surgery, Aster MIMS, Kasaragod, Kerala, India; 2 Department of Plastic Surgery, All India Institute of Medical Sciences, New Delhi, India

We tend to think of artificial intelligence (AI) as a futuristic prospect in medicine, yet in reality, it has already crept into our practice slowly and steadily. Patients are consulting AI before they walk through our doors, hospitals are turning to it to streamline daily operations, and there is an ever-growing body of literature on AI applications in all aspects of patient management. 1 2 Its reach extends further into digital health platforms, pharmaceutical companies, and health insurers, with startups mushrooming to offer symptom assessment, remote monitoring, personalized care pathways, and virtual care delivery. Free mobile apps are available for our patients to see what they would look like after a rhinoplasty, even while sitting at home. The truth is, *we are already in the era of AI* .

There is another truth we need to face. We, doctors, have limited knowledge of the technicalities of AI. That gap is perhaps the source of conflict in our minds. Is AI a magical tool that will empower us as clinicians, or will it lead to the deterioration of the critical thinking, imagination, and creativity that we have nurtured and honed over the years? How do we make the best of this tool without faltering in our duty towards patient safety and medical ethics? These questions trouble us and rightly so. But our hesitation to adapt cannot be permanent and restrictive, for as we debate, the technology continues to grow and surround us.

Plastic and Reconstructive Surgery is a specialty that has traditionally never shied away from adopting new technologies to solve problems and push boundaries.

In **preoperative planning** , deep learning algorithms can now automatically identify perforator vessels from computed tomography (CT) angiography studies, simplifying flap planning. 3 Machine learning models are being developed to predict complications in breast reconstruction, and some have been externally validated to augment standard clinical risk assessment. 4 5 6 AI-driven simulation tools are giving patients a visual language for their surgical goals, making consultations smoother. 7 **Intraoperatively** , AI-augmented robotic platforms are being used, powered with “computer vision” algorithms to analyze the surgical field in real time to identify vascular structures and reduce the risk of inadvertent injury. 8 9 Experimental systems are capable of assessing vessel quality under the microscope and guiding anastomotic technique in real time. 10

**Postoperatively** , AI-powered monitoring platforms can continuously track free flap perfusion, alerting surgeons to signs of vascular compromise hours before they would be detected conventionally by clinical observation. 11 Smart wound assessment tools allow patients to photograph their wounds at home with algorithms flagging concerning changes before the next scheduled visit. 12

Not all of this is ready for universal adoption, and the evidence base is still evolving. But we have an obvious insight: AI has several applications that could elevate the care we can provide to our patients, but the stakes are very high, so we must be cautious. *But how do we do that?*

That question has become considerably easier to answer now that India has begun to build the frameworks we need.

In February 2026, the Government of India published the **Strategy for Artificial Intelligence in Healthcare for India (SAHI)** with a vision to strengthen health care through the effective and responsible adoption of AI across all sectors. 13 It is a national framework that has laid the foundation for a pragmatic and enabling environment for wider adoption of AI in health care across the country.

SAHI addresses the hesitation for AI adoption we may have, due to its novelty. One of the seven guiding principles of SAHI is **Innovation over Restraint** . It guides us to choose the path of responsible innovation over that of cautionary restraint. We are expected to proceed with judgment, be more cautious about AI systems that have a more direct or critical impact on patient care, and be less stringent with those that are meant for administrative logistics.

Alongside SAHI, India also launched BODH, the **Benchmarking Open Data Platform for Health AI.** If SAHI is the policy framework, BODH is the ground-level infrastructure that makes responsible AI adoption actually possible. 14 BODH is built in collaboration with IIT Kanpur and the National Health Authority. It independently tests AI models against clinically representative Indian datasets across gender, geography, rural primary health centers, and tertiary hospitals. It produces a standardized evaluation report, which is especially important in a country like ours, where patient demographics and the availability of health care services vary widely. Our tools must be designed and validated for our population; otherwise, they can lead to dangerous bias. This is what BODH attempts to do.

SAHI also speaks of something exciting beyond the mechanics of governance. It tells us that India is not only a recipient of AI tools built elsewhere but also a driver of them. The digital health architecture that India has built under the Ayushman Bharat Digital Mission, with national health identifiers, interoperable records, and population-scale data infrastructure, provides a foundation for AI development that most countries are still trying to build.

*What does this mean practically for us as plastic surgeons? What must we do to ensure responsible adoption of AI?*

After giving this some thought, we propose that, as a community, we should go a step beyond mere adoption. We must aim for *AI stewardship.*

Adoption is passive, meaning we receive something built elsewhere and integrate it into our workflow. Stewardship is active and means taking responsibility for how a tool is introduced, used, monitored, and when it should be set aside.

We need to take charge simply because we are the ones accountable. SAHI is direct on the matter of accountability: **The surgeon stays accountable** . The responsibility for acting on any AI output rests with the clinician. To be fully accountable, we need to have knowledge and understanding of each tool we use. Just as we have built our surgical accountability on understanding anatomy, physiology, biochemistry, microbiology, pharmacology, and pathology, for the responsible use of AI, we require a genuine understanding of what the system is doing and where it might be wrong.

Here are some ways we can achieve AI stewardship:

- Asking the right questions and knowing the tools well before we inculcate them into our practice. We must be aware of who built the algorithm and on what data it was trained. We need to be aware whether that data included patients who look like ours, with skin types, body habitus, nutritional profiles, and comorbidities representative of our clinical population. We must understand both the advantages and shortcomings of the tools before we choose to use them.
- Participating in policy-making: Though SAHI states that the clinician is accountable, this oversimplifies the nuances of this delicate matter. We must actively ponder, debate, and ensure our representation in the framing of AI policy, in coordination with the government. For this, we need to have our groundwork and our internal committee ready.
- We need specialty-specific guidelines, and we need to write them ourselves. This should cover guidelines for AI-assisted simulation and preoperative visualization, the disclosure obligations to patients when AI has shaped their care, and the use of generative AI in advertising and social media. If we do not set these standards ourselves, we may be swayed by commercial or market forces. 15
- Another area of contribution for us as a community are data. For plastic surgery, the data needed to build useful AI models are very specific and very visual. Multicenter, anonymized registries of reconstructive outcomes, scar assessments, cephalometric data, and longitudinal aesthetic results are the raw material from which trustworthy, India-validated AI tools will eventually be built. BODH is the infrastructure waiting to receive precisely that data, while SAHI has laid the governance framework for sharing it responsibly. The registries we build today become the benchmarks that protect our patients tomorrow, and they position our specialty as a contributor to India's AI future rather than a passive consumer of tools developed elsewhere.
- To realize the goal of effective and ethical AI use, if there is one area that demands the greatest urgency, it is training. SAHI understands the need to build a trained workforce and calls for integrating AI competency into medical education. The National Board of Examinations in Medical Sciences (NBEMS) launched an AI training program in January 2026. We need to consciously train ourselves and our residents to evaluate AI tools critically. 16 Our Continuing Medical Education (CME) and conference scientific committees should take this into account during their planning.
- Then, there is another aspect to training. Residents graduating over the next few years will train and practice in a world where AI-assisted surgical planning is routine. If we are not careful about how we train them, we risk producing surgeons who perform well within the AI environment but struggle when the system fails. Deskilling in tasks delegated to automation is something we are all aware of, and we need to actively safeguard our specialty from it. Our specialty must be aware of and be able to adapt to the changes that are needed in the resident training with the growing introduction of AI in practice.

The opportunity in front of us is real, and we have every reason to be optimistic. India has built, in SAHI and BODH, a policy and validation framework that most countries are still trying to piece together. As a specialty, we have the opportunity to contribute our data to make the tools better for our patients, shape governance so that the rules reflect clinical reality, train our residents to be intelligent and critical users and to demand independent validation before tools enter our wards.

But none of that happens by itself. It will happen if we choose to engage seriously, train deliberately, demand accountability, and take responsibility. That is not a burden but something that our specialty has always done with every new technology that we have embraced. It is a challenge we must overcome to ride the new wave of AI, rather than be swallowed by it.

## References

[1] Kiwan O Al-Kalbani M Rafie A Hijazi Y Artificial intelligence in plastic surgery, where do we stand? JPRAS Open 2024 42 234 243 39435018 10.1016/j.jpra.2024.09.003 PMC11491964

[2] Arkoubi A Y The intelligent lift: Artificial Intelligence's growing role in plastic surgery - a comprehensive review Front Surg 2025 12 1.640588E6 10.3389/fsurg.2025.1640588 PMC12361134 40837783

[3] Lim B Cevik J Seth I Evaluating artificial intelligence's role in teaching the reporting and interpretation of computed tomographic angiography for preoperative planning of the deep inferior epigastric artery perforator flap JPRAS Open 2024 40 273 285 38708385 10.1016/j.jpra.2024.03.010 PMC11067004

[4] O'Neill A C Yang D Roy M Sebastiampillai S Hofer S OP Xu W Development and evaluation of a machine learning prediction model for flap failure in microvascular breast reconstruction Ann Surg Oncol 2020 27 09 3466 3475 32152777 10.1245/s10434-020-08307-x

[5] Naoum G E Ho A Y Shui A Risk of developing breast reconstruction complications: A machine-learning nomogram for individualized risk estimation with and without postmastectomy radiation therapy Plast Reconstr Surg 2022 149 01 1e 12e 34758003 10.1097/PRS.0000000000008635

[6] Braun S E Sinik L M Meyer A M Larson K E Butterworth J A Predicting complications in breast reconstruction: Development and prospective validation of a machine learning model Ann Plast Surg 2023 91 02 282 286 37489971 10.1097/SAP.0000000000003621

[7] Eldaly A S Avila F R Torres-Guzman R A Simulation and artificial intelligence in rhinoplasty: A systematic review Aesthetic Plast Surg 2022 46 05 2368 2377 35437664 10.1007/s00266-022-02883-x

[8] Riddle E W Kewalramani D Narayan M Jones D B Surgical simulation: Virtual reality to artificial intelligence Curr Probl Surg 2024 61 11 101625 39477664 10.1016/j.cpsurg.2024.101625

[9] Ward T M Mascagni P Ban Y Computer vision in surgery Surgery 2021 169 05 1253 1256 33272610 10.1016/j.surg.2020.10.039

[10] Tang M Sugiyama T Takahari R Assessment of changes in vessel area during needle manipulation in microvascular anastomosis using a deep learning-based semantic segmentation algorithm: A pilot study Neurosurg Rev 2024 47 01 200 38722409 10.1007/s10143-024-02437-6

[11] Kim J Lee S M Kim D E Development of an automated free flap monitoring system based on artificial intelligence JAMA Netw Open 2024 7 07 e2424299 39058486 10.1001/jamanetworkopen.2024.24299 PMC11282448

[12] Kitaguchi D Takeshita N Hasegawa H Ito M Artificial intelligence-based computer vision in surgery: Recent advances and future perspectives Ann Gastroenterol Surg 2021 6 01 29 36 35106412 10.1002/ags3.12513 PMC8786689

[13] Ministry of Health and Family Welfare Government of India Strategy for Artificial Intelligence in Healthcare for India (SAHI) 2026

[14] Kumar CS P View of BODH: Benchmarking Open Data Platform for India Health AI—A review of architecture, evaluation methodology, and implementation framework for clinical AI validation in India Int J Latest Technol Eng Manag Appl Sci 2026 XV 1324 1334. Accessed May 24, 2026 at:https://www.ijltemas.in/submission/online/article/view/4224/5700

[15] Kenig N Monton Echeverria J Rubi C Ethics for AI in plastic surgery: Guidelines and review Aesthetic Plast Surg 2024 48 11 2204 2209 38456892 10.1007/s00266-024-03932-3

[16] Kapila A K Hamdi M Time to evolve plastic surgery education? Integrating robotic techniques and artificial intelligence into training curricula Plast Reconstr Surg Glob Open 2024 12 05 e5778 38699284 10.1097/GOX.0000000000005778 PMC11062664

